# Tactile Decoding of Edge Orientation With Artificial Cuneate Neurons in Dynamic Conditions

**DOI:** 10.3389/fnbot.2019.00044

**Published:** 2019-07-02

**Authors:** Udaya Bhaskar Rongala, Alberto Mazzoni, Marcello Chiurazzi, Domenico Camboni, Mario Milazzo, Luca Massari, Gastone Ciuti, Stefano Roccella, Paolo Dario, Calogero Maria Oddo

**Affiliations:** ^1^Scuola Superiore Sant'Anna, The BioRobotics Institute, Pisa, Italy; ^2^Department of Linguistics and Comparative Cultural Studies, Ca' Foscari University of Venice, Venice, Italy

**Keywords:** force and tactile sensing, neuro-robotics, conduction delays, mechanoreceptors, cuneate neurons, biologically-inspired robots, spiking neural networks

## Abstract

Generalization ability in tactile sensing for robotic manipulation is a prerequisite to effectively perform tasks in ever-changing environments. In particular, performing dynamic tactile perception is currently beyond the ability of robotic devices. A biomimetic approach to achieve this dexterity is to develop machines combining compliant robotic manipulators with neuroinspired architectures displaying computational adaptation. Here we demonstrate the feasibility of this approach for dynamic touch tasks experimented by integrating our sensing apparatus in a 6 degrees of freedom robotic arm via a soft wrist. We embodied in the system a model of spike-based neuromorphic encoding of tactile stimuli, emulating the discrimination properties of cuneate nucleus neurons based on pathways with differential delay lines. These strategies allowed the system to correctly perform a dynamic touch protocol of edge orientation recognition (ridges from 0 to 40°, with a step of 5°). Crucially, the task was robust to contact noise and was performed with high performance irrespectively of sensing conditions (sensing forces and velocities). These results are a step forward toward the development of robotic arms able to physically interact in real-world environments with tactile sensing.

## Introduction

As robots become more accepted to be part of our daily social and work environments, the research focus has taken a diversion toward more human centric design and learning paradigms. Many research studies in recent time have taken inspiration from nature and its evolutionary principles, to exploit the robustness and low computational costs in performing a dynamic task in un-trained surroundings (Ijspeert, 2014).

**Figure 1:**
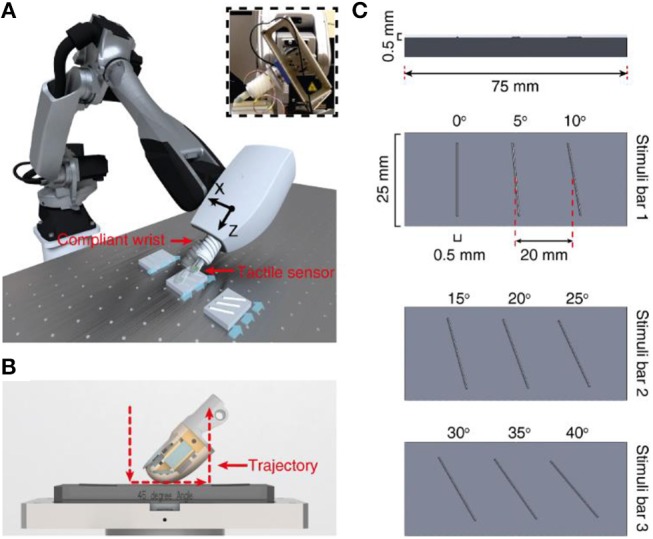
Methods. **(A)** Robot setup with finger and compliant wrist integration. Insert demonstrates the integration of tactile sensor onto robot end effector with the help of a compliant wrist. **(B)** Illustration of the active touch protocol, where the finger is moved across the stimuli (red dotted line). **(C)** Representation of all the 9 ridge stimuli (3D printed with a height and width of 0.5 × 0.5 mm, with placement of three ridges).

In the last few decades several neurophysiological studies in mammals focused on understanding the role of the various families of mechanoreceptors (sensory receptors that are sensitive to mechanical distortions) spread across the human skin, and their role in projecting information about external world to brain (Johansson and Flanagan, 2009; Abraira and Ginty, 2013). Such studies, subsequently led to enhanced understanding of the profile of sensory information, that neuronal circuits receive during simple object manipulation tasks. These studies describe the nature of spatiotemporal information (spiking responses) in tactile sensory afferents. Further, there is evidence that tactile feature extraction can happen already at afferent stages (Johansson and Flanagan, 2009; Gollisch and Meister, 2010; Weber et al., 2013), complementing with central information processing in the sensory cortex (Bensmaia et al., 2008a; Hsiao, 2008). Explicit studies on the effects of geometric features such as edge orientations (vertical line tilted in an angle, Figure 1C) on sensory afferents reported about information processing at peripheral stages of sensory perception (Pruszynski and Johansson, 2014). In alike manner, in the present study our research focuses on developing a robust tactile perception for robots, based on bioinspired paradigms. Toward this goal, we aimed at reproducing the intelligence embodied in the connectivity of the peripheral human tactile sensory system. By reproducing this connectivity structure in our robotic system we mimicked the hardwired architecture that has been hypothesized in humans (Johansson and Flanagan, 2009). In particular, we captured the features of the conduction delays along the neural pathways from peripheral to the central processing stages, to allow the tactile processing of geometric features such as edge orientations.

Many research studies in recent time have created neuro-robotic systems by combining computational models based on various neurophysiological data (Saal et al., 2017) along with tactile sensors to reconstruct tactile afferent like responses (Lee et al., 2017; Rongala et al., 2017; Osborn et al., 2018). Recently, biomimetic computational models of peripheral tactile perception were extended to take into account the second neuronal layer of decoding (Bologna et al., 2013; Rongala et al., 2018a,b) and this feature was also used for edge detection (Hay and Pruszynski, 2018). Some robotic studies have also focused on geometric feature extraction techniques, where Ruben and colleagues (Ponce Wong et al., 2014) studied edge orientation with similar approach of robotic-arm based exploration and classification. They used support vector regression methods to learn and classify the stimuli, which requires offline training. One other study from Hernandez and colleagues (Martinez-Hernandez et al., 2013) conducted static edge perception experiments by tapping the stimuli with tactile sensor to demonstrate passive tactile perception for contour following. This approach used a probabilistic classifier based on Bayesian formalism for tactile perception. The edge detection was done only on right angles (0°, 90°, 180°, 270°) with controlled sensing force.

One characteristic feature (Ijspeert, 2014) of all the aforementioned studies is that they were conducted under controlled sensing conditions. However, in order to build biomimetic devices that are able to process tactile information in the real world, we need to capture the way this process is robust to varying sensing conditions. Therefore, here we adopted a neuro-inspired paradigm to create a tactile feature extractor and verified it to be an effective information decoding strategy. Combining a two-layer neurocomputational model based on discrete events and delays, along with soft robot interfaces led us to develop a functional tactile system, that was able to deliver effective decoding of geometric edge orientations under varying sensing conditions (sensing forces and velocities). We have also assessed that our system performance was robust to variation in sensing forces.

## Materials and Methods

### Tactile Sensor

**Figure 2:**
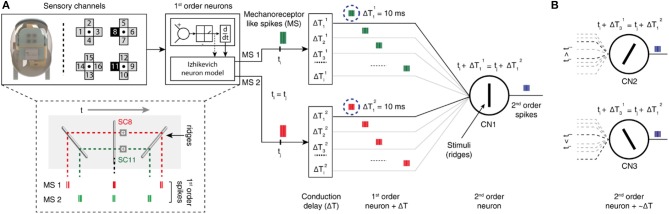
Two-layer neuron architecture. **(A)** (*Left*) fingertip structure and arrangement of 16-sensory channels. The data from two sensory channels (8 and 11) are injected as input current into the Izhikevich neuron model (1st order neurons), that deliver mechanoreceptor-like spike trains. (*Below*) representative illustration of mechanosensors responses [MS1 and MS2 (corresponding to SC8 and SC11 respectively)] to ridges having an angle of −45°, 0°, and 45° relatively to the direction orthogonal to stimulus-finger relative motion. (*Right*) schematic diagram of second order neuron implementation for edge orientation processing. Two different mechanosensors inputs (green and red spike trains, MS1, and MS2) for a single stimulation sequence converge to the same cuneate nucleus neuron (CN_i_, 2nd order neuron), through specific conduction delays along the pathway. In the illustrated example, CN1 generates output spikes (blue spike trains) only when it receives MS inputs that coincide. **(B)** The other CNs (CN2 and CN3) respond to different angle stimuli, depending on the set of conduction delays and subsequent temporal relations formed between the inputs.

**Figure 3:**
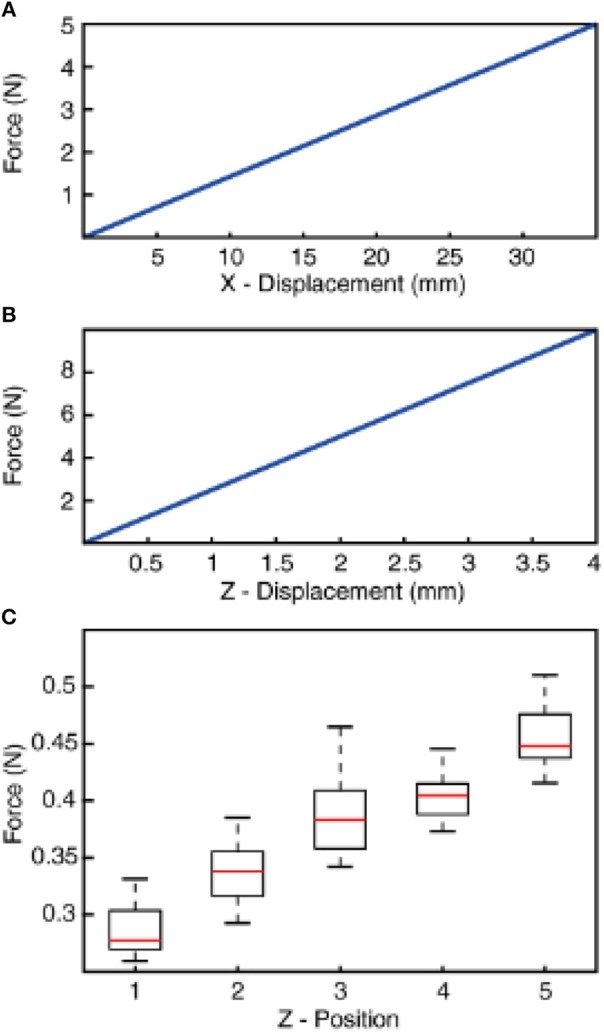
Normal and shear stiffness of the compliant wrist and resulting sensing force between the tactile sensor and stimuli. **(A)** Tangential stiffness (across x-axis) of the compliant wrist displacement as the function of applied force. **(B)** Compressive stiffness (across z-axis) of the compliant wrist displacement as the function of applied force. **(C)** Sensing forces recorded during finger contact with all stimuli (1–9) associated to the different z-axis positions of the robot (Z-positions 1–5). The z-positions are obtained with a progressive increase by a step of 0.5 mm toward the stimuli from home position (as described in Methods). The boxplot illustrates the range of force across 5 mechanical repetitions and all 9 stimuli. The forces are recorded using a loadcell that is placed between the compliant wrist and the robotic arm.

For this research study we used a tactile fingertip, with a core element of MEMS (Micro Electro-Mechanical System) sensor. This sensor comprises of 2 × 2 piezoresistive sensor array (Figure 2A), arranged with 2.36 mm pitch [SensorPitch (SP) in Equation (1)]. Each sensor array comprises of four sensory channels (four piezoresistors, with cross-shape arrangement), constituting for a total of 16 sensory-channels in a 22.3 mm^2^ area (Beccai et al., 2005; Oddo et al., 2011). This tactile sensor demonstrated sensitivity for both tangential and normal forces (Oddo et al., 2011) and precision and repeatability in the neuromorphic encoding-decoding of a varied range of stimuli that include ridges (Oddo et al., 2016) and naturalistic textures (Rongala et al., 2017). In this research study we consider data from 2 sensory channels [sensory channel 8 (SC8) and sensory channel 11 (SC11), Figure 3A], that are sensitive to the tangential force arising along the stimulus sliding direction. These channels are also space shifted along same axis with respect to the stimulus direction, which makes them appropriate to validate the conductional delay hypothesis. We convert this analog sensory information to neuromorphic spikes (event-based representation of data alike in neurons) using neuron models that are described in further sections of this article.

### Compliant Wrist

A compliant wrist was assembled on a rigid end effector of a 6 DoFs anthropomorphic robot (Comau Racer-7-1.4) through a loadcell (6-axis Nano 43, ATI Industrial Automation, Apex, USA). Whereas, the other end of compliant wrist carries the tactile sensor (Figure 1A). The design of the wrist was aimed at realizing a soft joint, enabling adaptation upon contact (between the fingertip and the external surfaces). Such a compliant element also prevents damage to the tactile sensor without affecting its sensitivity. The structure of the compliant wrist was shaped as a cylinder with a diameter and height of 40 × 60 mm. Moreover, the soft wrist was realized with helicoidally flextures to increase its flexibility. The joint was manufactured through molding of a polymeric viscous material, namely Dragon Skin (10 medium, Smooth-on, USA). Two metallic plates were used on the top and bottom surfaces of the cylinder, as a mechanical interface between the robot end-effector and the tactile sensor. Under the typical loads for this specific application (in the order of 1 N), analyses with a finite element model (COMSOL Multiphysics, COMSOL Inc., USA) showed a compressive stiffness of 2.5 N/mm and a stiffness of 0.14 N/mm along the tangential directions (Figures 3A,B).

### Stimuli

**Figure 4:**
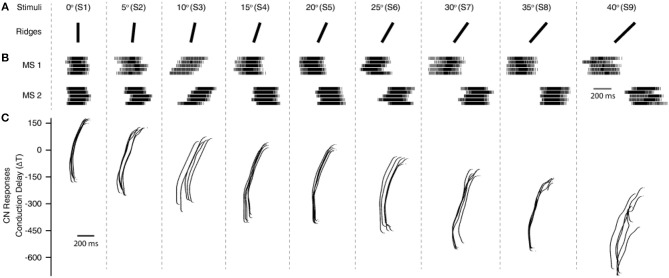
Processing stimuli orientation. **(A)** Representation of all 9 stimuli, with ridge angles ranging from 0 to 40° with a step of 5°. **(B)** Artificial mechanosensors spike train responses (MS1 and MS2, 1st order neurons) encoded using Izhikevich neuron model based on the tactile sensor data input (MS1 and MS2 responses associated to the analog data from sensory channel 8 and 11, SC8 and SC11 from Figure 1). The five spike train responses for each mechanoreceptor, show the 5 experimental repetitions for each stimulus. **(C)**, Cuneate neurons (CNs, 2nd order neurons) responses for all 9 stimuli (S1–9), across a range of differential conduction delays varying between 175 and −700 ms, with a step of 1 ms. The illustrated responses of MSs and CNs are for a single sensing force (Z1 from Figure 3C) and sensing velocity (5 mm/s) of the experimental protocol.

We used 9 rectangular shaped ridges as stimuli with dimension of 0.5 × 0.5 mm (height and width). Each of this ridge is fabricated with an inclination angle, ranging from 0 to 40° angles with a step of 5° (Figures 1C, 4A). These ridges were fabricated using 3D printing technology. For these experiments, we printed three ridges with consecutive angles onto a single stimuli bar, resulting in total 3 stimuli bars bearing the 9 stimuli (as shown in Figure 1C). As these ridges are printed on three different stimuli bars, they have slightly varied physique because of different bending and other small deformations that occur in stimuli due to the 3D printing technology. This further pose additional challenges in the generalization abilities of the developed neuro bioinspired architecture.

### Experimental Protocol

We adopted an active touch protocol, where the stimuli are kept fixated on a rig and the robot end-effector (hooked with tactile fingertip) is maneuvered across the surface of the stimuli (as illustrated with blue arrows in Figure 1A; Supplementary Video). The robot is controlled using a real-time industrial controller (IC3173, programmed with NI LabVIEW, LabVIEW Real-Time and LabVIEW FPGA, along with C5G COMAU robot controller), that received position control commands. The robot default home position was put just above (along z-axis of the robot) the surface of the tactile stimuli. Further, the robot end-effector was moved along its z-axis (toward the stimuli), until a reference sensing force (force exerted between the tactile fingertip and stimuli) of ~200 mN was reached. This is considered as the initial z-axis position (Z1, in Figure 3C) for all the stimuli.

Once the “Z1” position reached, keeping the z-axis of the robot locked, the fingertip was slid across the surface of stimulus (robot end-effector translated in cartesian space) for a length of 15 mm with a fixed sliding velocity, covering the whole surface of the ridged stimuli. The fingertip was held still for 1 s in this position, at the end of sliding. Finally, the fingertip was retracted away from the stimulus along the z-axis. This trajectory (Figure 1B, red dotted line) was maintained across all the ridges. Once the sliding was finished across all three ridges, the robot was brought back to the home-position. The robot was progressed 0.5 mm in z-axis (toward the stimulus) from the previous z-axis position (Z1–4) to generate varied sensing/contact forces, but without implementing precise force-feedback control. This experimental protocol was repeated 5 times across each stimulus, in-order to assess the repeatability of the system. In the following study we have presented and validated the neuro-inspired architecture for five different sensing forces (Figure 3C, with *P* < 0.001, using ANOVA test) and five different sensing velocities (5, 10, 15, 20, and 25 mm/s).

### Neuron Model (Mechanoreceptors, 1st Order Neurons)

The tactile sensor analog data was fed as virtual current input (*I*_*input*_) to a custom implementation of the Izhikevich neuron model (Izhikevich, 2003) to generate the mechanosensors-like spatiotemporal spike responses (Figures 3A, 4B, 1st order neurons). The Izhikevich neuron model was chosen in order to reproduce the adaptation dynamics, which is an important characteristic that is observed in mechanoreceptors (Johansson and Flanagan, 2009). The basic Izhikevich neuron model was defined by the following nonlinear differential equations, where *v* is membrane potential and *u* is the adaptation variable.

(1) $$\begin{array}{l} \dot{v}=Av^{2}+Bv+C-u+\frac{I_{input}}{C_{m}}\\ \dot{u}=a(bV-u) \end{array}$$

When the membrane potential reached the spike threshold of *30 mV*, an output spike was produced followed by a reset,

(2) $$\begin{array}{r} if\;V\geq 30\;mv,\;then\;\{\begin{array}{c} v\;\leftarrow c\\ u\;\leftarrow u+d \end{array} \end{array}$$

*A, B, C* are the standard Izhikevich model parameters, whereas the *a, b, c*, and *d* parameters were chosen as specified in Table 1, to reciprocate regular spiking behavior.

**Table 1:** Izhikevich neuron model parameters.

**A**	**B**	**C**	**C_m_**	**a**	**b**	**c**	**d**
0.04*s*^−1^*V*^−1^	5 *s*^−1^	140 *Vs*^−1^	1F	0.02 *s*^−1^	0.2	−65 mV	8 mV

### Neuron Model (Cuneate Neurons, 2nd Order Neurons)

The cuneate neurons (CNs) were also modeled as regular spiking Izhikevich neurons, based on the similar differential equations described above (Equations 1, 2). Whereas, the input current (*I*_*input*_)to the CNs was modeled as the summation of current-based post-synaptic potential(*PSP*_*tot*_, Equation 4) (Cavallari et al., 2014) from mechanosensor neurons along with addition of specific differential conduction delays (ΔT) (Figure 3). At each given spike time of mechanosensor output ($t_{i}^{*}$) is converted to a single *PS*P_*i*_, who's kernel was given by Equation (3).

(3) $$\begin{array}{l} PSP_{i}=\frac{\tau_{l}}{\tau_{d}\;-\;\tau_{r}}\;\times \;[\exp\left(-\frac{t-\;\tau_{l}-\;t_{i}^{*}}{\tau_{d}}\right)\\ \;-\;\exp\left(-\frac{t-\;\tau_{l}-\;t_{i}^{*}}{\tau_{r}}\right)] \end{array}$$

(4) $$\begin{array}{r} PSP_{tot}=\sum_{i\;\in pre}PSP_{i} \end{array}$$

The parameters, decay time (*τ*_*d*_), rise time (*τ*_*r*_) and latency time (*τ*_*l*_) defines the shape of the *PS*P_*i*_ kernel. The basic configuration values *τ*_*d*_ = 12.5 ms, *τ*_*r*_ = 4 ms, and *τ*_*l*_ = 21 ms (constant to calculate the ratio) are chosen based on the previous assumptions of calcium concentration induced in the synapse as presented in our previous work (Rongala et al., 2018b). $t_{i}^{*}$ gives the input spike-time from *i*^*th*^ mechanosensor.

### Conduction Delays

A conduction delay is the time step (Δ *T*) that was added to the whole output spike-train of mechanoreceptors (1st order neurons) along the afferent pathway. These delays bear a resemblance to the conduction times in nerves that connects tactile afferents (in hand) to the cuneate neurons (in brainstem) of humans (Johansson and Flanagan, 2009). We tested differential conduction delays ranging from 175 ms (MS1 ahead of MS2) to −700 ms (MS2 ahead of MS1) with a step of 1 ms, constituting for 876 conduction delays.

### Classification Algorithm

Given the characteristics of the data we have chosen a linear discrimination method trained with supervised learning. The classifier was trained and tested using a 5-fold cross-validation, which was repeated for 100 iterations to ensure the robustness of the classifier and training procedure. We have taken advantage of the inbuilt MATLAB® functions to perform this computation.

**Figure 5:**
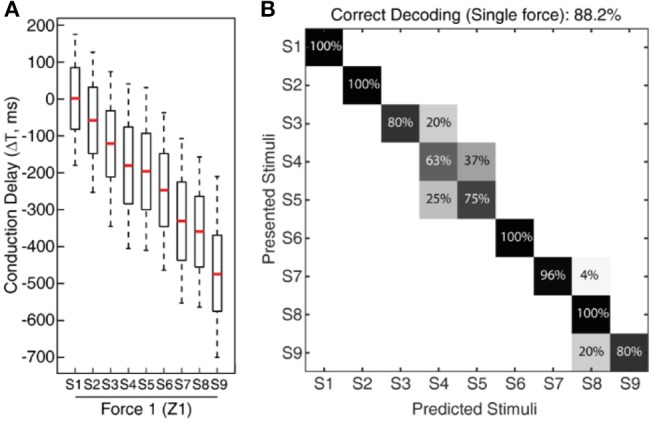
Classification of CNs responses across all 9 stimuli for fixed sensing force. **(A)** Boxplot illustrating the spiking probability for CNs responses (Figure 4C) across all the nine stimuli for a given force. The boxplot demonstrates a gradual shift in the CNs spiking respective to the stimuli angle and conduction delay. The spiking probability is calculated across 5 experimental repetitions of each CN. **(B)** Confusion matrix demonstrates a decoding performance achieved across all the 9 stimuli (for a fixed sensing force (Z1) and sensing velocity (5 mm/s)) based on CNs responses, using supervised linear discrimination classifier. The decoding accuracy was 100% across 5 stimuli with a step size of 10° (S1, S3, S5, S7, and S9), and 88.2% with a step size of 5°.

**Figure 6:**
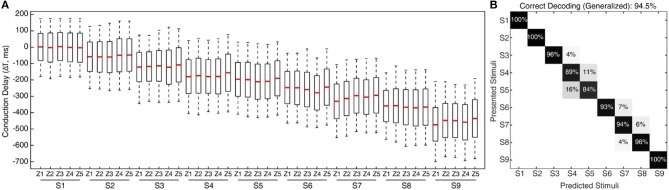
Classification of CNs responses irrespective of sensing forces. **(A)** Spiking probability across all the nine stimuli and five different sensing forces (Figure 3C), for each conduction delays. These CNs responses are for a single sensing velocity (5 mm/s). This plot clearly demonstrates almost similar spiking probability, robust to the variation of the sensing force, thus supporting generalization ability of the proposed approach. **(B)** Confusion matrix illustrating the decoding performance achieved by CNs irrespective of forces (labeling stimuli irrespective of sensing force), across all the 9 stimuli. The decoding accuracy was 100% across 5 stimuli with a step size of 10° (S1, S3, S5, S7, and S9), and 94.5% with a step size of 5°.

**Figure 7:**
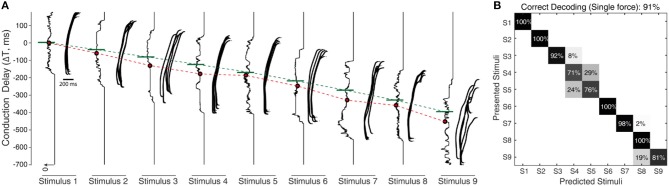
Processing stimuli orientation with noise. **(A)** Raster plot illustrates the responses of CNs (2nd order neuron) for all 9 stimuli (S1–9), across a range of fixed conduction delays varying between 175 and −700 ms, with a step of 1 ms. The responses illustrated are for fixed sensing force and sensing velocity. Each CN model is simulated for 5 experimental repetitions with an addition of 19 noise repetitions each, constituting a total of 100 repetitions for each CN configuration. The noise was generated by a gaussian distribution with σ = 10 ms. The continuous plot alongside each raster plot illustrates the density of spiking for that respective differential conduction delay. The green line illustrates the theoretical value (Equation 5) of conduction delay. The median value of the density is indicated in red dot. The red line across stimuli illustrates the linear shift of spiking probability across different conduction delays. **(B)** Confusion matrix illustrating the decoding performance that is achieved by CNs across all the 9 stimuli (for a single sensing force and sensing velocity), using supervised linear discrimination classifier. The decoding accuracy was 100% across 5 stimuli with a step size of 10° (S1, S3, S5, S7, and S9), and 91% with a step size of 5°.

**Figure 8:**
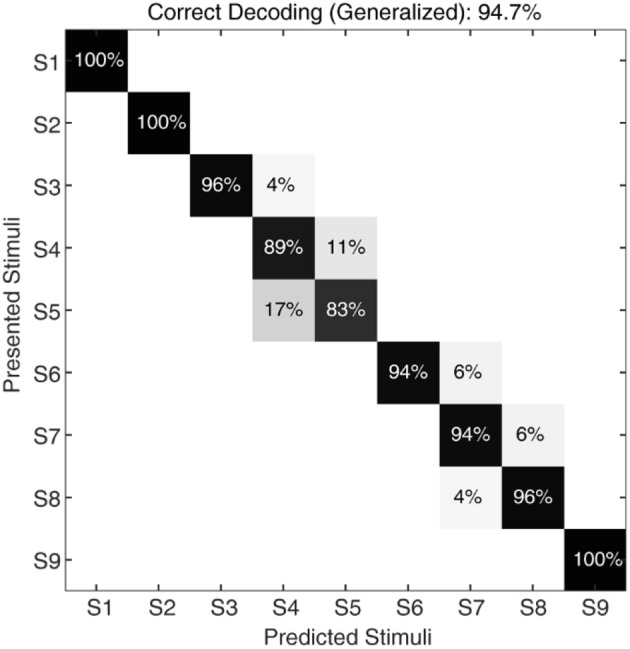
Classification of CNs responses with noise, irrespective of sensing forces and for a given sensing velocity (5 mm/s). The confusion matrix illustrating the generalized decoding performance achieved by CNs irrespective of sensing force, across all the 9 stimuli. The decoding accuracy was 100% across 5 stimuli with a step size of 10° (S1, S3, S5, S7, and S9), and 94.7% with a step size of 5°.

A probability density of the CN spike responses is calculated using the histogram function in MATLAB®, with a binsize of 10. Further, the median of this probability distribution was chosen as an input vector to the above described classifier (one-dimensional input). While considering single force-based decoding (Figures 5B, 7B, 10A), the input vector data is binned as 9 classes, representing all the 9 stimuli. The same followed for generalized decoding (Figures 6B, 8, 10B), the input vector data was binned as 9 classes (9 stimuli), irrespective of sensing forces.

## Results

To explore the possibility and the efficacy of a biomimetic approach to tactile sensing, we developed an experimental set-up comprising a biomimetic tactile sensor (Oddo et al., 2011) linked to a customized soft wrist and 6 degree-of-freedom robotic arm (Figure 1A). This interaction led to the efficient classification of stimuli containing 9 angle ridges selected in a range from 0° to 40° with a step of 5° (Figure 1C) based on the contact with the biomimetic fingertip (Figure 1C).

A two layer neuro-computational model was used in processing these tactile sensory information. In the first layer we make use of the Izhikevich neuron model to convert the output of the 16-channel tactile sensor data to multiple neuron spiking responses (Figure 2A) (Rongala et al., 2017). This allowed mimicking the response properties of human mechanosensors (MSs). In the second layer, we emulate cuneate neurons (CNs), again as regular spiking Izhikevich neurons. The inputs to CNs were modeled as summation of current based post-synaptic potentials from the MSs (see Methods). The key of the decoding mechanism is the connectivity between the two layers. This connectivity embodies a model of event-based encoding of tactile responses (Figure 2A), emulating the discrimination properties of cuneate neurons (CNs) based on pathways with differential delay lines (Figure 2A). The CN is considered in this model as a coincidence detector (Johansson and Flanagan, 2009), meaning that a specific CN responds when there is a superimposition of input mechanosensor spike timing. Based on this hypothesis, each CN encodes a specific angle, depending on the match of the stimulus-driven delay between the activation of the MSs with the difference between their conduction delays, which results in a synchronized and thus effective stimulation of the appropriate CN recipient (Figure 2A). Thanks to this mechanism, the probability of each CN to respond to a certain stimulus depends of the combination of specific conduction delays connecting it to its presynaptic MSs (Figure 2A).

To validate this approach, initially we conducted the experimental protocol across all the 9 stimuli with a single sensing force (Z1, Figure 3C) and a single sensing velocity (5 mm/s, V1). Two sensory channels data (SC8 and SC11, Figure 2A, see Material and Methods for details) were selected as inputs to the neuronal processing to emulate mechanosensors-like responses (MS1 and MS2, Figure 4B). These spike trains were then fed into CNs, with a given conduction delay time (ΔT). The second layer was constituted by 876 different cuneate neurons, each identified by a specific conduction delay between the inputs received from MS1 to MS2. The conduction delays are ranged from 175 to −700 ms with a step size of 1 ms. Figure 4C show the responses of CNs based on the MS1 and MS2 inputs for a given differential conduction delay, across all stimuli. The CNs responses demonstrated in Figure 4C (raster plots) are for all 5 experimental repetitions. Further, in order to test the effectiveness of the designed system we analyzed the spike distribution probability across all the CNs for each stimulus (Figure 5A). The spiking distribution illustrated in Figure 5A, are based on the CNs responses shown in Figure 4C. We found a gradual shift in the median of the spiking probability across the range of conduction delays, as a function of the stimuli ridge orientation (Figure 5A). This shift in probability illustrates that certain CNs respond more to a specific stimulus, i.e., those whose differential conduction delay tends to compensate the theoretical latency (*T*_*Theory*_) between the spiking activation of the related MSs.

(5) $$\begin{array}{r} T_{Theory}=SP\times \frac{\tan\left(RA\right)}{V} \end{array}$$

Where, *SP* denotes the Sensor Pitch (see Methods), *RA* denotes the Ridge Angle (edge orientation angle of the stimuli), and *V* defines the sensing velocity. This relationship suggests that it might be possible to invert the process and decode the presented orientation, looking at the distribution of firing across the CNs population. This decoding strategy was inspired by that hypothesized in humans (Johansson and Flanagan, 2009), where the vast amount of information from 10,000's of tactile afferents across the hand is reduced into a small and useful sensory dimension, and then further transmitted to higher level cognitive processing. For validation of the information content in CN responses we used a linear classification technique (see Methods for details), which yielded 88.2% accuracy in decoding across all the 9 stimuli (Figure 5B, chance level 11.1%). As demonstrated in the confusion matrix (Figure 5B), in some cases only consecutive stimuli are confounded, which is also contributed by the little angle difference between two stimuli along with dynamic sensing conditions in real time robot operation. Whereas, we achieved 100% correct classification (for all 5 sensing forces) restricting the decoding across five stimuli, with stimuli angle variation of 10° (S1, S3, S5, S7, and S9). This led us concluding that the actual accuracy of our device is better than 10° ridge identification.

In the real world the sensing conditions might change, hence we wanted to test that the performance of our device was not restricted to a particular force of contact. For this generalization test we conducted experiments similar to the one described above, but with 5 different levels of target sensing forces and a fixed sensing velocity (5 mm/s, V1). To further stress the generalization ability, these 5 target force levels were not generated by precise force-feedback control, but by setting 5 different z-axis positions of the robot end-effector (Figure 3C, see Methods for details). We then computed the CNs spiking probability across all the 9 stimuli and 5 forces (Figure 6A). We grouped then across all the forces the median of spiking probability belonging to same stimulus and used it as the feature vector for classifier to validate the CNs responses across all stimuli irrespective of sensing forces. We attained 94.5% correct decoding across all the nine stimuli (Figure 6B), with a stimuli angle variation of 5°. This shows that high level of accuracy can be achieved independently of the variations of the sensing force. This highly-generalized decoding performance proves our encoding strategies to be robust to varying sensing forces, thanks to an architecture mimicking the intelligence embodied in the neural pathways from periphery to the brain.

To further stress the robustness of our neuromorphic device, we introduced temporal jitters (with a gaussian noise of σ = 10 ms) in each experimental repetition. This contributed 100 repetitions of each CN encoding for each stimulus and sensing force level. Figure 7A shows the responses of CNs to these 100 repetitions, along with the spiking density for each conduction delay (continuous plot beside raster plot). We observed a coherent phenomenon of gradual shift in spiking intensity across the range of conduction delays for each stimulus (Figure 7A, red dot depicting the mean of spiking intensity) even with additional temporal noise. We also observed that the mean spiking intensity across each stimulus (Figure 7A red dotted line) fell near to the theoretically estimated conduction delays (Figure 7A green dotted line) based on Equation (5). We then yielded a high decoding accuracy for these CN responses across all the 9 stimuli, even in the presence of the introduced temporal noise in MS spiking activity. In this condition with added spike jitter we attained a correct decoding between 91% and 100% across nine stimuli, for individual sensing force and fixed sensing velocity (Figures 7B, 10A). Further, we attained a decoding accuracy of 94.7% irrespective of sensing forces and fixed sensing velocity (Figure 8).

**Figure 9:**
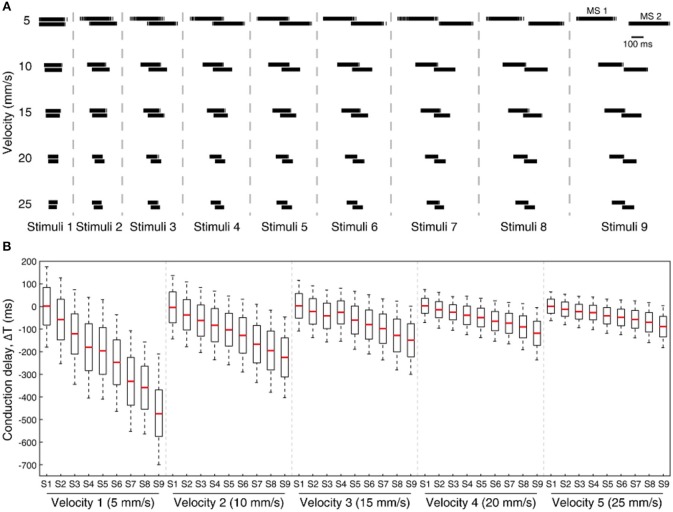
Processing stimuli orientation across varying sensing velocity for a fixed sensing force (Z1, Figure 3C). **(A)** Artificial mechanosensors (MS1 and MS2, 1st order neuron) spike train responses for all 9 stimuli, across 5 different sensing velocities. **(B)** Boxplot illustrates the probability of spiking across all the stimuli for 5 varying sensing velocities, for a range of conduction delays. The spiking probability is calculated on the CNs responses. Each CN model is simulated for 5 experimental repetitions with an addition of 19 noise repetitions each (gaussian distribution with σ = 10 ms), constituting a total of 100 repetitions for each CN configuration.

Further, we have also tested our system for five different sensing velocities (5, 10, 15, 20, and 25 mm/s) for a given sensing force. The mechanosensor spike responses (MS1 and MS2) demostrate a gradual shift in their spike timing with respect to the stimuli, along with homogenous transformation of total spike time with respect to the sensing velocities (Figure 9A). Further we analyzed the spiking probability of CNs responses for each stimulus and given sensing velocity (Figure 9B). We found a gradual shift in the median of the spiking probability across the range of conduction delays (Figure 9B), showing that some CNs are sensitive to a specific stimulus, under an optimal sensing velocity.

**Figure 10:**
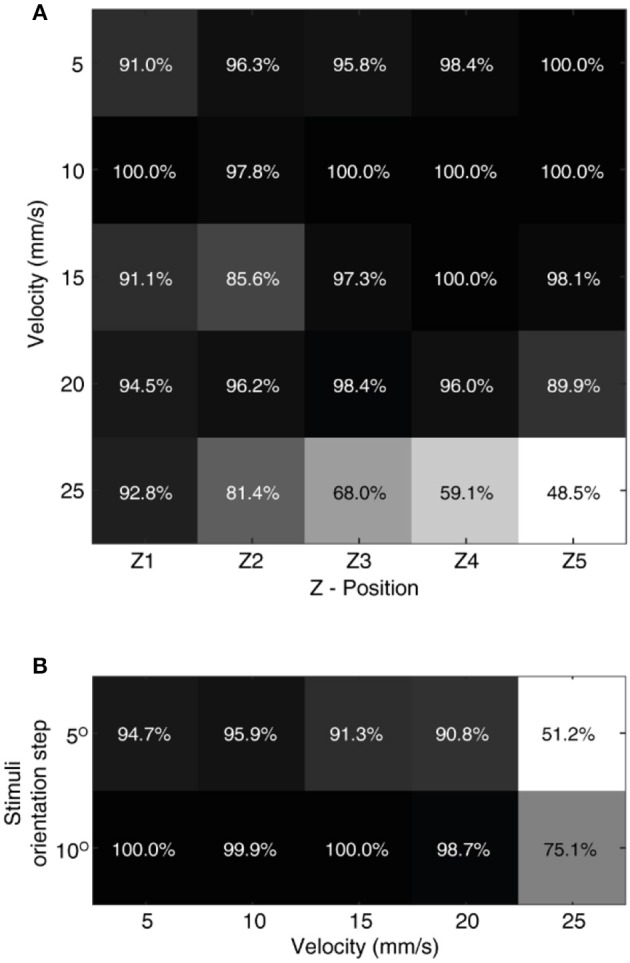
Effect of sensing dynamics on decoding. **(A)** The decoding performance achieved by CNs neurons across all the 9 stimuli, for a given combination of sensing force and sensing velocity. The confusion matrix for each given decoding performance is illustrated in Supplementary Figure 1. **(B)** Generalized decoding performance (irrespective of sensing force), achieved by CNs for a given sensing velocity. The decoding accuracy was presented across all 9 stimuli (S1–S9) with a step size of 5°, and across 5 stimuli with a step size of 10° (S1, S3, S5, S7, and S9).

In order to evaluate the effect of these dynamic sensing conditions on our neuromorphic device, we have performed stimulus classification based on the CNs responses (with additional temporal jitter in the MSs) for a combination of all 5 sensing forces and 5 sensing velocities. We achieved more than 90% correct decoding in 20 out of 25 experimental conditions (Figure 10A). A low decoding performance was observed at high sensing velocities with high sensing forces, where the spiking probability boundaries overlap. Further, we attained a high decoding performance irrespective of sensing force, for each sensing velocity (Figure 10B). This high decoding accuracy proved that the proposed architecture was highly robust to noise and dynamic sensing conditions.

## Discussion

We developed an artificial tactile system, with a bioinspired tactile sensor mounted onto a traditional 6 degrees-of-freedom industrial robot using a compliant wrist, that allowed adaptation to irregular sensing dynamics present in the surrounding tactile world. Further, we used a neuroinspired two-layer architecture to process the tactile sensory information. Thanks to such a synergy, we achieved an excellent orientation decoding performance (100% for 10° and 94.9% across 5° orientation step, for stimuli ranging from 0° to 40°). This is an excellent result as compared to the state-of-art orientation detection in robotic applications (Martinez-Hernandez et al., 2013; Ponce Wong et al., 2014). Taking advantage of the precisions in existing tactile sensors and computational systems, and combining them with biomimetic architectures can lead to building functional systems that are more capable in sensing when compared to the psychophysical studies that report about 20° angular perceptual threshold in humans (Bensmaia et al., 2008b). Further, exploiting neuromorphic hardware systems to build spiking neuronal networks across the population of sensors enabled a computationally efficient implementation of a functional tactile system. Moreover, we were able to capture another peculiarity of human tactile detection, i.e., to perform decoding in a way largely irrespective of sensing forces, across different sensing velocities.

The multiple experimental sensing conditions, the irregularities of 3D printed stimuli, the intrinsic limitations in robot precision along with soft compliance in wrist, created a versatile sensing condition that generated a variety of sensory responses. The tactile system presented in this research, capable to cope with such realistic variability in experimental conditions, plays a keen role in robotic applications. Such biomimetic approach will allow the robots to adapt and perform effectively irrespective of the continuously changing environments.

Note that in this research study we considered nerve conduction delays that are larger than what have been observed in mammals (Johansson and Flanagan, 2009). In this study what we were interested into was to reproduce the principle of differential delay matching the encoding of the stimulus orientation. As the pitch of our tactile sensors was much larger than in humans, where each hand is densely populated with 10,000s of tactile sensors afferents, we used proportionally larger conduction delays coherently with the prediction of Equation (5). Anyway, the used sensor density allowed achieving a high decoding across the varied set of stimuli considered, providing evidence that robotics technology can benefit of neuroscientific advances and in turn robotics science can contribute to investigating neurophysiological hypotheses (Yang et al., 2016).

## Author Contributions

UR, AM, and CO conceived and designed the study, analyzed data, discussed the results, and wrote the paper. UR and MC performed experiments. UR, MC, DC, MM, LM, GC, SR, PD, and CO developed the experimental setup. UR implemented the cuneate-based model. CO ideated the cuneate-based model, supervised, and coordinated the study. All authors contributed to manuscript revision, read and approved the submitted version.

### Conflict of Interest Statement

The authors have a pertinent patent application pending. The authors declare that the research was conducted in the absence of any commercial or financial relationships that could be construed as a potential conflict of interest.
