# Supplementary material for: Tactile Decoding of Edge Orientation With Artificial Cuneate Neurons in Dynamic Conditions
Source: Front Neurorobot. 2019 Jul 2;13:44. doi: 10.3389/fnbot.2019.00044 (PMC6614200; doi:10.3389/fnbot.2019.00044)
Supplement: Supplementary file 2 [file Data_Sheet_1.docx]

Supplementary Material

Tactile Decoding of Edge Orientation with Artificial Cuneate Neurons in Dynamic Conditions

**Udaya Bhaskar Rongala^1,2*^*,* Alberto Mazzoni^1^, Marcello Chiurazzi^1^, Domenico Camboni^1^, Mario Milazzo^1^, Luca Massari^1,2^, Gastone Ciuti^1^, Stefano Roccella^1^, Paolo Dario^1^, Calogero Maria Oddo^1*^**

*** Correspondence:**U. B. Rongala and C. M. Oddo
[udayabhaskar.rongala@santannapisa.it](mailto:udayabhaskar.rongala@santannapisa.it) and [calogero.oddo@santannapisa.it](mailto:calogero.oddo@santannapisa.it)

#
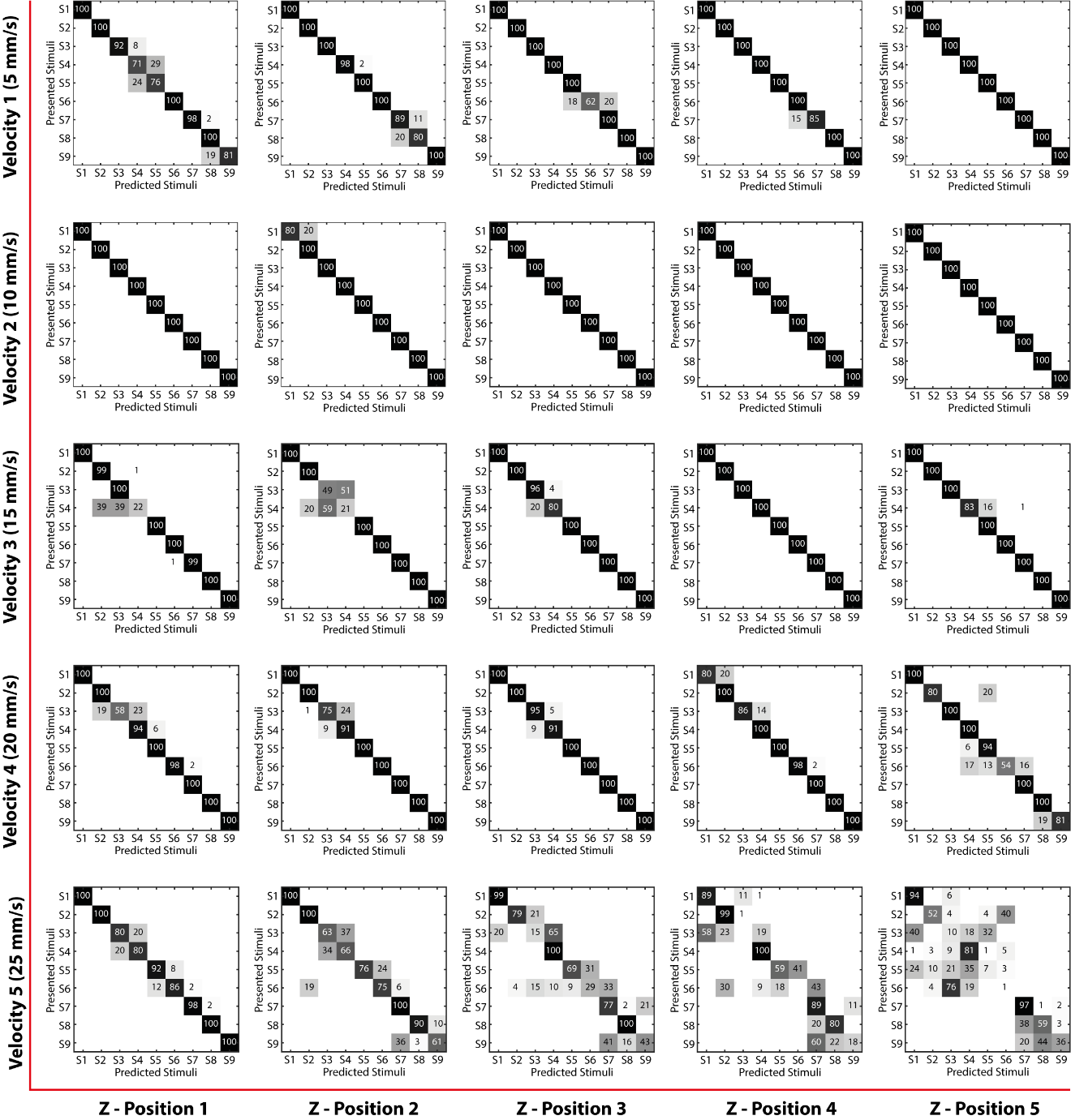
Supplementary Figures

**Supplementary Figure 1.** Confusion matrix demonstrating the decoding performance achieved across all the 9 stimuli, for a combination of 5 sensing forces (Z-Position 1 to 5) and 5 sensing velocities experimental conditions (Velocity 1 to 5).
